# Posterior-only vertebral column resection for revision surgery in post-laminectomy rotokyphoscoliosis associated with late-onset paraplegia: A case report and literature review

**DOI:** 10.1097/MD.0000000000005690

**Published:** 2017-01-10

**Authors:** Youping Tao, Jigong Wu, Huasong Ma

**Affiliations:** Department of Orthopedic Surgery, the 306th Hospital of People's Liberation Army (PLA), Beijing, China.

**Keywords:** paraplegia, posterior vertebral column resection, post-laminectomy rotokyphoscoliosis, revision

## Abstract

**Rationale:**

Severe post-laminectomy spinal deformity associated with late-onset paraplegia is a complex and rare disorder. Little is known about revision surgery in post-laminectomy rotokyphoscoliosis associated with late-onset paraplegia treated by the single stage posterior-only vertebral column resection (VCR) procedure.

**Patient concerns and diagnoses:**

The patient was a 14-year-old male diagnosed as post-laminectomy rotokyphoscoliosis associated with late-onset paraplegia. He underwent posterior total laminectomy through the thoracic spine for intramedullary spinal cord tumors at the age of 3 years in another hospital. He then developed kyphosis deformity 1 year after laminectomy, and underwent posterior spinal fusion without instrumentation at 9 years of age. However, the deformity gradually progressed over the years. Seven months before admission to our hospital, he developed a significant progression of neurological deficits, including weakness of strength and sensation in lower extremities bilaterally, with no bladder or bowel dysfunction. There was no improvement of spinal cord function with conservative measures, and he required a wheelchair for movement.

**Interventions:**

The patient underwent posterior-only VCR by single stage with the purposes of spinal cord decompression and spinal deformity correction.

**Outcomes:**

Postoperatively, he was transferred to the intensive care unit (ICU) and required positive pressure ventilation support to improve his respiratory condition. The child experienced cerebrospinal fluid leak (CSF) which resulted in an unplanned return to the operating room. The neurological function improved from preoperative Frankel C to Frankel D within 12 months of surgery, and recovered completely to Frankel E by 18 months. At the 24 month follow-up, the good neurological function was maintained; pulmonary function tests (PFTs) revealed improved forced vital capacity (FVC) and forced expiratory volume for 1 second (FEV1). The patient's coronal major curve and sagittal kyphosis were corrected from 70° to 21°, and 170° to 75°, respectively.

**Lessons:**

These findings demonstrated that single-stage posterior-only VCR is efficacious but challenging for revision surgery in post-laminectomy rotokyphoscoliosis associated with late-onset paraplegia.

## 1 Introduction

Spinal cord tumors are not common and account for 1 to 10% of all pediatric central nervous system tumors.^[1–3]^ Most intramedullary spinal cord tumors require surgical treatment as early as possible.^[4]^ Spinal deformity following posterior laminectomy for spinal cord tumors in children has been reported by a number of studies.^[5–14]^ When the spinal deformity is corrected with a plaster cast or only posterior fusion without instrumentation, the deformity would exhibit progression and require revision operation.^[7]^ In 2012, Duman et al^[10]^ first reported a case of post-laminectomy rotokyphoscoliosis causing late-onset paraplegia. However, the patient declined surgical intervention, and no neurological status improvement was obtained.

It is widely accepted that limited correction may be provided without osteotomy in severe spinal fusion deformity.^[15]^ Posterior spinal osteotomy procedures, such as Smith Peterson, Ponte, and pedicle subtraction osteotomy (PSO) may not yield a great amount of deformity correction in very severe spinal deformity cases.^[15,16]^ The vertebral column resection (VCR) technique may provide an effective option to correct these severe and rigid spinal deformities with limited flexibility. The conventional VCR procedure can be performed by 2-stage anterior and posterior vertebral column resection, fusion, and segmental spinal instrumentation.^[17,18]^ In 2002, Suk et al^[19]^ first introduced posterior-only VCR, which was popularized by Lenke's team.^[20]^ Compared with combined anterior and posterior VCR, the posterior-only VCR technique is a single procedure and obviates the need to open the thoracic cavity, with additional negative effects on pulmonary function. More recently, several investigators have reported experiences with posterior-only VCR for severe and rigid spinal deformity, in both primary and revision patients.^[15,21–27]^

However, to the best of our knowledge, studies applying posterior-only VCR for revision surgery in postlaminectomy rotokyphoscoliosis associated with late-onset paraplegia are scarce. We here present a rare case of postlaminectomy rotokyphoscoliosis associated with late-onset paraplegia, who successfully underwent revision surgery by single-stage posterior-only VCR.

## 2 Consent

Written informed consent was obtained from the patient's parents on behalf of the child, for the publication of this case report and any accompanying images. A copy of the written consent is available for review by the editor of this journal.

## 3 Case report

A 14-year-old male was admitted to our clinic for treatment of extremely severe spinal deformity associated with progressive late-onset paraplegia. His medical history included the diagnosis of intramedullary astrocytoma at the age of 3 years in another hospital, where he underwent posterior total laminectomy through the thoracic spine (from T4 to T8), with tumor excision performed without fusion. Intramedullary astrocytoma represents 29% of pediatric spinal cord tumors in the literature.^[28]^

**Figure 1:**
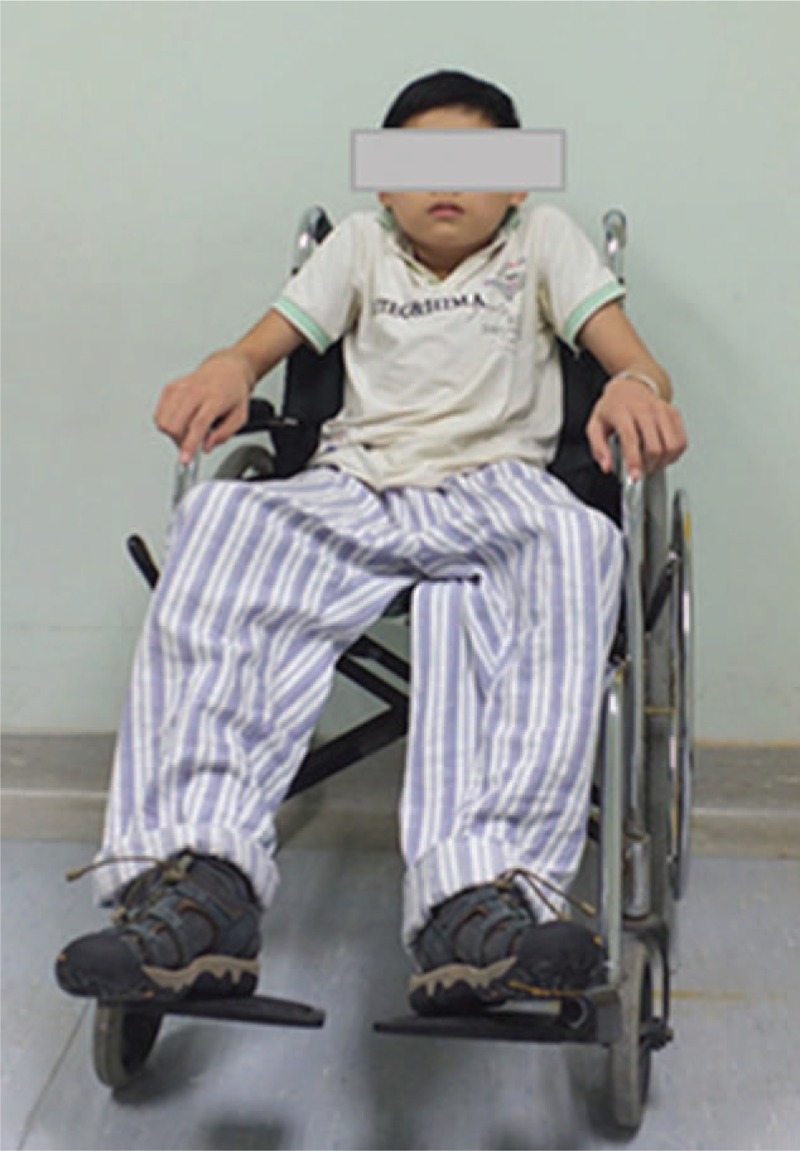
The patient could not walk independently and required a wheelchair for movement.

He reported that kyphosis deformity began to appear 1 year after posterior laminectomy. Later, he underwent posterior spinal fusion without any instrumentation at 9 years of age. However, the deformity gradually progressed over the years. Seven months before admission to our hospital, the patient began to feel lack of strength and sensation in lower extremities bilaterally, with no complains regarding bladder or bowel dysfunction. No improvement in spinal cord function was achieved with conservative measures, and the patient gradually lost the ability to walk and required a wheelchair for movement (Fig. 1). The patient and his family denied any recent or remote history of trauma, tuberculosis, or any other infections. The family history was unremarkable; meanwhile, the patient exhibited signs of serious psychosocial problems since he had to use a wheelchair in daily life.

**Figure 2:**
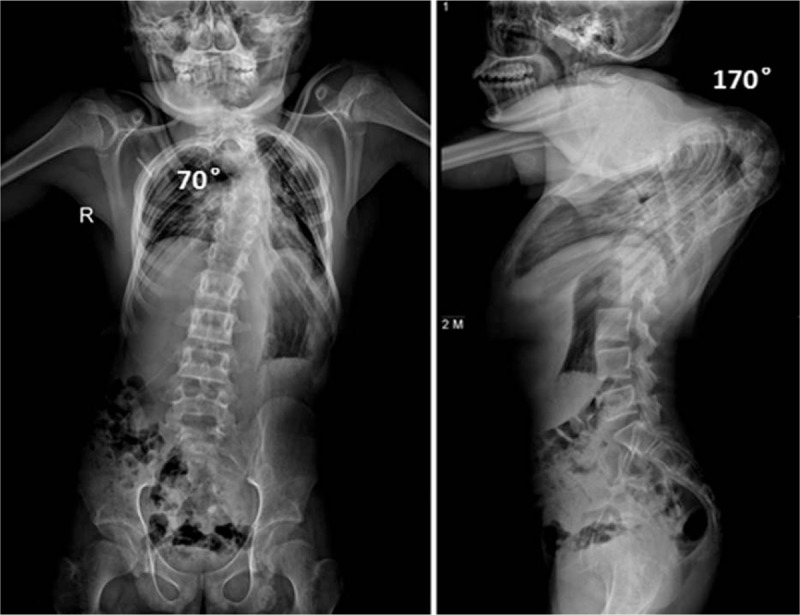
Preoperative scoliosis radiography showed coronal and thoracic kyphosis Cobb's angles of 70° and 170°, respectively.

**Figure 3:**
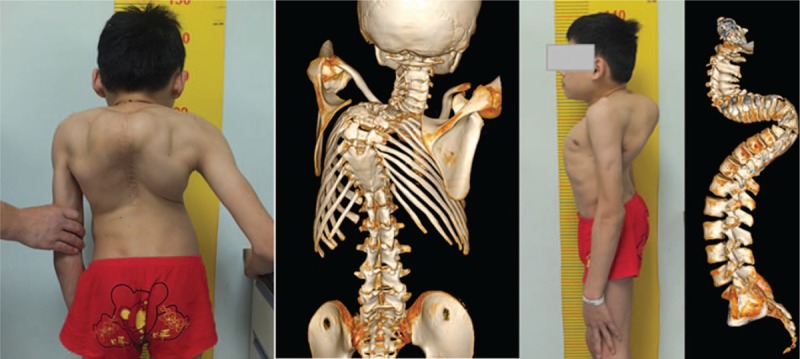
Preoperative 3D CT scans showing extremely severe postlaminectomy thoracic kyphotic deformity and posterior spinal fusion. 3D CT = three-dimensional-computed tomography.

**Figure 4:**
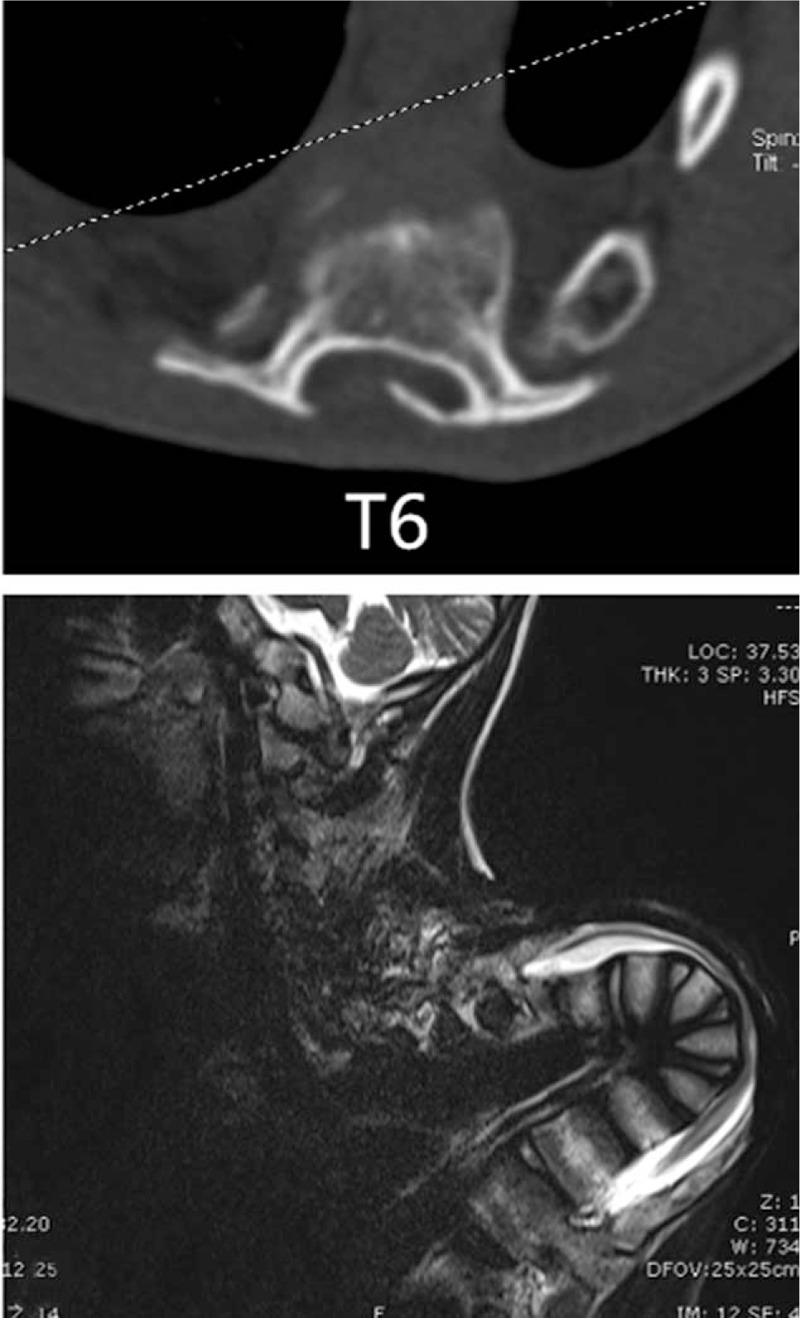
Magnetic resonance imaging (T2-weighted) and CT scans of the spinal canal revealing spinal cord compression at the apex of the kyphosis in the thoracic spine (T6). CT = computed tomography.

The patient's preoperative coronal and sagittal thoracic kyphosis Cobb's angles were 70° and 170°, respectively (Fig. 2). Preoperative whole spine three-dimensional-computed tomography (3-D CT) scans showed extremely severe postlaminectomy thoracic kyphotic deformity; with the apex at T6 vertebra and previous spinal fusion (Fig. 3); magnetic resonance imaging (MRI) of the spinal canal revealed spinal cord compression at the apex of the kyphosis (Fig. 4). There was no neurological abnormality such as split cord malformation or tethered cord. The electrodiagnostic investigation demonstrated neurological deficit. Pulmonary function tests (PFTs) revealed forced vital capacity (FVC) and forced expiratory volume for 1 second (FEV1) of 31.8% and 33.8%, respectively. Cardiac Doppler ultrasonographic investigation demonstrated mitral and tricuspid regurgitation, with no anomalies of other visceral organs.

Physical examination showed skin hypoesthesia below the T6 level and lower limb spasticity. The patient had weakness of the intrinsic musculature of lower limbs, with left and right lower extremity Grade 3/5 and Grade 1/5 motor strength in all muscle groups, respectively. His Achilles and knee tendons showed hyperreflexia, and the Babinski sign was positive. The overall spinal cord function was classified as Frankel C.

**Figure 5:**
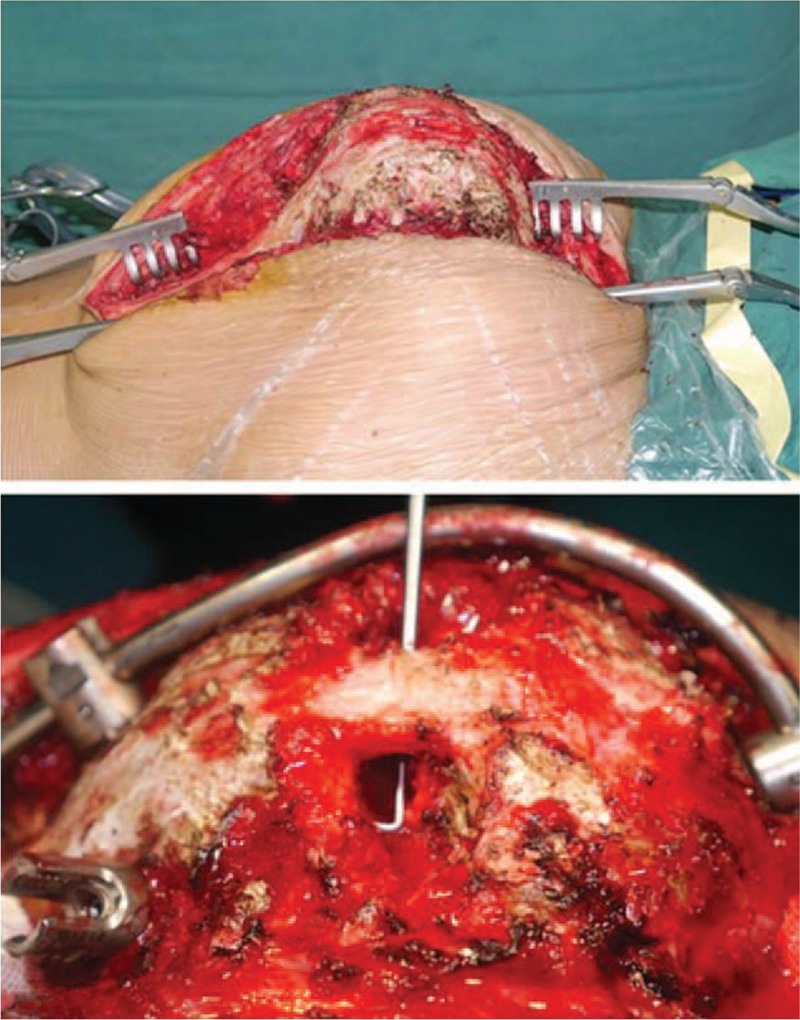
Intra-operative photograph showing posterior vertebral column resection (T6) performed and posterior spinal fusion with pedicle screw fixation from T2 to L3.

In 2014, the patient underwent preoperative assessment by a team of pediatrics, neurosurgery, anesthesiology, and respiratory specialists, and diagnosis of postlaminectomy rotokyphoscoliosis associated with late-onset paraplegia and severe pulmonary ventilation disorder were confirmed based on the findings. The patient was suggested a revision surgical treatment, and the single stage posterior-only VCR procedure was scheduled. In brief, pedicle screws were inserted using the free-hand technique based on previous studies.^[29,30]^ The patient underwent a VCR at T6 for spinal cord decompression, deformity correction, and posterior spinal fusion from T2 to L3 (Fig. 5). The autograft and allograft were used for posterior final spine fusion. During the operation, motor evoked potential (MEP), somatosensory evoked potential (SEP), and wake up test were performed to assess intraoperative complications of spinal cord injury.

The operation time was 450 minutes, and blood loss was 1500 mL. Intraoperatively, a dural tear was noted and repaired; MEP and SEP signals were lost during the entire operation, and the wake-up test was positive. Postoperatively, the patient was transferred to the intensive care unit (ICU) and required positive pressure ventilation support to improve his respiratory condition. On postoperative day 2, he was extubated and transferred to the orthopedic ward, where he received neurotrophic medicines and rehabilitation exercises. The patient required a chest tube for postoperative pleural effusion. The child experienced unplanned return to the operating room for cerebrospinal fluid leak (CSF) on postoperative day 18. The plastic thoracolumbosacral orthosis braces were preserved for 6 months after revision operation.

**Figure 6:**
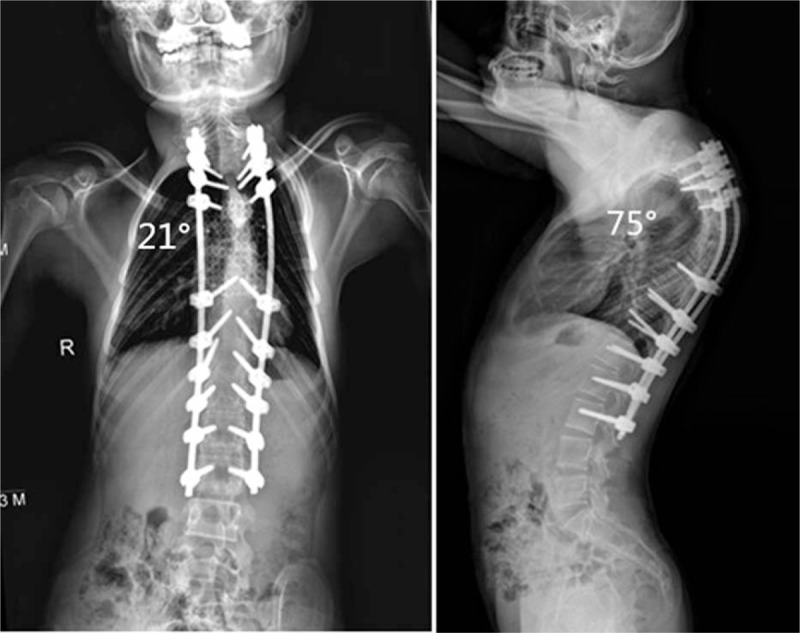
At the final follow-up, long-standing scoliosis radiographs revealed the coronal main curve was corrected to 21° and sagittal kyphosis curve to 75°.

**Figure 7:**
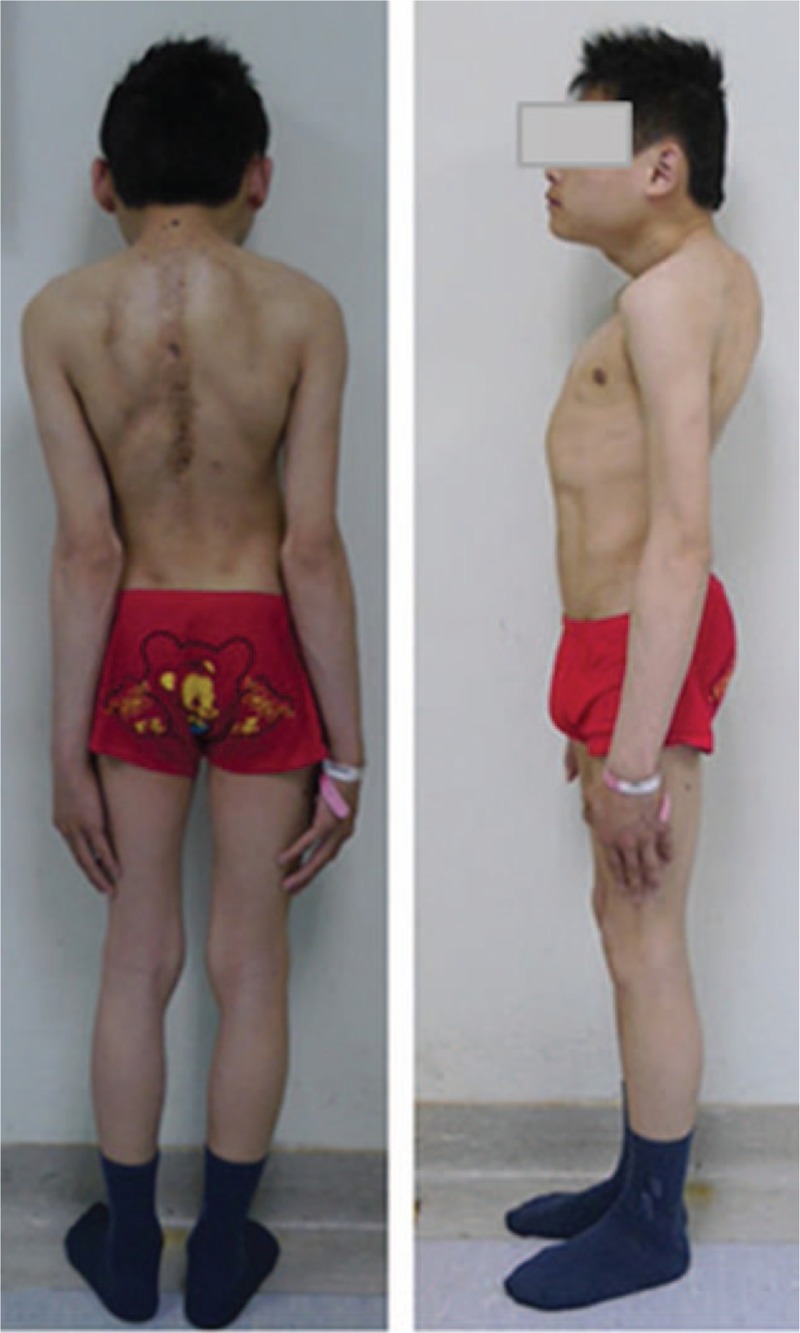
Clinical photographs showing overt improvement at the final follow-up.

The spinal cord function improved gradually after the posterior-only VCR procedure. At 3 months, left and right intrinsic musculature of lower limbs both exhibited Grade 3/5 motor strength in all muscle groups. Left and right intrinsic musculature of lower limbs recovered to Grade 4/5 motor strength at 12 months postoperatively. At 18 months postsurgery (follow-up visit), intrinsic musculature of lower limbs displayed Grade 5/5 motor strength, and sensation in lower extremities bilaterally had returned to normal, with the neurological status improving from preoperative Frankel C to Frankel E. At the 24-month follow-up, the good spinal cord function was maintained. Long-standing scoliosis radiographs demonstrated that the major curve at coronal plane was improved to 20° immediately after surgery and 21° (correction rate, 70%) at final follow-up; the thoracic sagittal kyphosis curve was improved to 76° immediately after surgery and 75° (correction rate, 55.9%) at the final follow-up (Fig. 6). PFTs revealed FVC and FEV_1_ of 40.5% and 43.5%, respectively. Postoperative clinical photographs showed overt improvement (Fig. 7).

## 4 Discussion

Several reports have described spinal deformity as a common complication after laminectomy to remove spinal tumors. In 1965, Tachdjian and Matson^[13]^ reported spinal deformities in 26% of infants and children treated with laminectomy for intraspinal tumors. In 2005, de Jonge et al^[7]^ reported that of 76 children with malignant tumors treated with laminectomy or laminoplasty and/or radiation therapy, 67 developed late-onset spinal deformity. Spinal deformities following laminectomy can be prevented by surgical and nonsurgical treatments; however, when corrected with only posterior fusion without instrumentation, post-laminectomy spinal deformities may progress during the period of rapid growth in children.^[3]^ The present patient had undergone posterior spinal fusion without instrumentation, with spinal deformity gradually progressing over the years; the boy developed paraplegia due to severe postlaminectomy spinal deformity.

In 2005, Suk et al^[22]^ reported the use of posterior-only VCR in 16 patients with severe and rigid spinal deformity (scoliosis more than 80°); mean preoperative scoliosis of 109.0° was corrected to 45.6°, with a correction rate of 59%. In 2009, Lenke et al^[20]^ reported that posterior-only VCR is a safe but challenging technique for severe primary or revision pediatric spinal deformities (58% coronal correction and 53% sagittal correction). In the present case, coronal and sagittal correction rates were 70% and 55.9%, respectively.

Revision surgical treatment for severe and rigid spinal deformity poses challenges to spine surgeons, due to the absence of bony landmarks, high risks of neurological deficit, and high blood loss or long surgery time.^[30]^ Glassman et al^[31]^ showed that patients are more likely to have complications if they had undergone previous spine surgery. Previous studies described revision surgical treatments for severe pediatric or adult deformity that were performed with staged posterior procedures or combined anterior/posterior surgeries.^[23,32,33]^ Although staged or combined anterior/posterior procedures for revision surgeries are effective, staged procedures expose patients to anesthetic risks and operation can be performed more than once; in addition, the anterior/posterior spinal fusion could open the thoracic cavity, with further negative effects on pulmonary function. The current findings revealed that single stage posterior-only VCR can achieve a similar correction rate for post-laminectomy rotokyphoscoliosis associated with late-onset paraplegia.

Suk et al^[22]^ reported that posterior-only VCR provides adequate chance for spinal canal decompression. The present patient underwent posterior-only VCR for spinal deformity correction and spinal cord decompression at the apex of kyphosis. Postoperatively, although he experienced major surgical complications, including pleural effusion, CSF, sensation and intrinsic musculature in lower extremities bilaterally gradually improved to normal. He has been able to walk independently and participate in normal daily activities with a high degree of satisfaction.

In 2004, Bumpass et al^[34]^ reported small but statistically significant increases in absolute pulmonary measures in pediatric patients after posterior-only VCR, especially those with angular kyphosis most strongly associated with improved postoperative pulmonary function. In the present case, postoperative PFTs revealed that FVC and FEV1 were overtly improved.

The strength of this article is that after single stage posterior-only VCR with no anterior approach for revision surgical treatment of postlaminectomy rotokyphoscoliosis associated with late-onset paraplegia, the neurological status improved from preoperative Frankel C to Frankel E. Postoperatively, the child experienced improvement of pulmonary function, and overt coronal and sagittal correction rates. As limitations, the retrospective nature of this study and the relatively short follow-up should be mentioned. Longer follow-up is warranted to assess postoperative clinical and radiographic results.

## 5 Conclusion

Overall, our results revealed that posterior laminectomy for treatment of spinal cord tumors in childhood can lead to spinal deformity. When the patient undergoes posterior spinal fusion without instrumentation, spinal deformity may gradually progress during the periods of fast growth. Extremely severe postlaminectomy thoracic kyphotic deformity is relatively complex and rare, and could lead to serious neurological complications, including paraplegia. Single-stage posterior-only VCR might be an efficacious but challenging option for revision surgery in postlaminectomy rotokyphoscoliosis associated with late-onset paraplegia.
