# Posterior-only vertebral column resection for revision surgery in post-laminectomy rotokyphoscoliosis associated with late-onset paraplegia: A case report and literature review: Erratum

**DOI:** 10.1097/MD.0000000000006930

**Published:** 2017-05-12

In the article, “Atypical presentation of paroxysmal nocturnal hemoglobinuria treated by eculizumab: A case report”,^[1]^ which appeared in Volume 96, Issue 1 of *Medicine*, The corresponding author information appeared by mistake as “Correspondence: Jigong Wu, igong Wu, Department of Orthopedic Surgery, the 306th Hospital of People's Liberation Army (PLA), Beijing 100101, China (e-mail: docwjg@126.com).” It should have appeared as “Correspondence: Jigong Wu, Department of Orthopedic Surgery, the 306th Hospital of People's Liberation Army (PLA), Beijing 100101, China (e-mail: docwjg@126.com).”
